# Chemical Speciation and Potential Mobility of Heavy Metals in Forest Soil Near Road Traffic in Hafir, Algeria

**DOI:** 10.5696/2156-9614-11.30.210614

**Published:** 2021-05-28

**Authors:** Fatima Zahra Benhachem, Djamila Harrache

**Affiliations:** 1 Department of Hydraulics, Institute of Sciences and Technology, University Center of Maghnia, Algeria; 2 Laboratory for the Application of Electrolytes and Polyelectrolytes Organic (LAEPO), Abou Beker Belkaid University, Algeria; 3 Department of Chemistry, Faculty of Exact Sciences, University of Djillali Liabes, Sidi Bel Abbes, Algeria

**Keywords:** heavy metals, soil, road traffic, Hafir, speciation

## Abstract

**Background.:**

Different fractions of metals, of varying origin and reactivity, are present in sediments of forest soil. Forest ecosystems are privileged sites for the deposition of persistent organic pollutants carried by the atmosphere.

**Objectives.:**

The present study describes the current state of metallic contamination around the Hafir forest, located southwest of Tlemcen, Algeria, based on analysis of total sediment mineralization and the speciation of each metal to examine the effects of emissions due to road traffic.

**Methods.:**

The distribution and migration of ten heavy metals were studied, including cadmium (Cd), nickel (Ni), cobalt (Co), chromium (Cr), manganese (Mn), lead (Pb), copper (Cu), zinc (Zn), iron (Fe), and silver (Ag), and four major elements: potassium (K), sodium (Na), magnesium (Mg), and calcium (Ca) in the superficial horizon of forest soil (0–20 cm) at different road distances (0–1700 m), in two sampling campaigns in the dry and wet seasons around the Hafir forest, Algeria.

**Results.:**

Bioavailability appears to be relatively low due to the small amount of metals present in the carbonate fraction and the alkaline pH. The impact of road traffic was observed in the variability of the concentrations of several trace elements in forest soil, such as Co, Mn, Ni, Zn, Pb, Ag, Cd. They were generally observed at very high levels along the roadside due to dry atmospheric deposition.

**Conclusions.:**

The high levels of metals in the carbonate, reducible and residual phases indicate a direct influence on the environment stemming from road traffic near the forest as well as contamination from rainfall in the area.

**Competing Interests.:**

The authors declare no competing financial interests.

## Introduction

Metals are inorganic micropollutants naturally present in soils, originating from the parent rock on which they were formed, most often in mobile form but at very low concentrations. The accumulation or mobilization of these micropollutants depends on several factors associated with the type of soil and element. As a result of atmospheric deposition resulting mainly from industrial and agricultural activities, road traffic and fires, metallic contamination of soils explains, in surface horizons, the current contents of metallic trace elements (MTE) such as cadmium (Cd), copper (Cu), zinc (Zn) and lead (Pb).^1^ Atmospheric pollution results from industrial (plant discharges) and urban activities (exhaust gas, etc.). It is important to distinguish the diffused air intakes of distant origin and the more concentrated localized inputs in assessments of atmospheric pollution. Diffuse intakes include dust and aerosols derived from heaters as well as the automobile engines. Localized inputs result from accidental anthropogenic intakes related to industrial activities without effective protection against dispersion in the environment.^2^ Metallic contamination of soils due to previous or current local atmospheric deposits explains the current contents of MTE such as Cd, Cu, Zn and Pb.^1^ Among these metallic elements, some (Cu and Zn) function as micronutrients at low levels but become toxic at high concentrations.^3^ Others such as Pb or Cd are toxic at low concentrations and can constitute a serious public health problem because of bioaccumulation.^4^ In soils, metals are naturally present in relatively unmovable forms and at low levels. Elements such as mercury (Hg), silver(Ag), Pb, Cu, nickel (Ni), Zn and Cd are naturally present in soil and are generally the result of alteration of the underground mother rock.^5^ Determination of the total element concentration, although insufficient to describe these environmental processes, is carried out in order to estimate the importance of the elements present from a quantitative viewpoint.^6^ Total concentration provides only partial information for the prediction of the behavior of metallic elements in the environment, hence the need to determine element speciation.^7^ Toxicity of a metal depends on its speciation (chemical form) as much as on environmental factors.^8^ Chemical speciation has been used in different contexts for their identification and quantification, and to describe the processes responsible for the distribution of species and their reactivity.^9^ Each species possesses its own chemical characteristics and will react differently depending on the environment. The chemical speciation of a metal depends on its oxidation state, its interaction with the other compounds of the system (clays, organic matter, oxyhydroxides) and environmental conditions (pH).^10^,^11^

In order to estimate the mobility of heavy metals, chemical agents are used to isolate and extract metals associated with certain phases identified in soil.^12^ This extraction is then followed by measurement of the concentration of the extracted metals in the solution. These tests are primarily used in approaches of risk analysis or transfer to the food chain.^13^–^16^ There are two main types of extraction. Specific extraction, targeting a fraction of the soil,^17^ relies on the use of a single extractant, which may be water, an organic solvent (ethanol), a neutral salt (potassium chloride (KCl), magnesium chloride (MgCl_2_), calcium nitrate (Ca(NO_3_)_2_), potassium nitrate (KNO_3_), a weak acid (acetate, oxalate), a complexing agent (ethylenediaminetetraacetic acid (EDTA), diethylene triamine pentaacetic acid (DTPA)) or a strong acid which is slightly concentrated (hydrochloric acid (HCl), nitric acid (HNO_3_)).^18^,^19^

Sequential extraction involves several chemical agents (generally from three to eight), performed by chaining the extractions on the same sample (sequentially) to determine the splitting (speciation) of the metallic elements within the main constituents of the soils. After Tessier's work on river sediments,^20^ the elements are generally separated into five fractions: exchangeable, related to carbonates, linked to (hydr) oxides, bound to organic matter, and residuals, trapped in the silicates. However, Tessier's sequential extraction scheme is often inadequate and many authors have modified the list of chemical agents used.^21^–^23^

**Abbreviations**
*AFNOR*: Association Française de Normalisation
*MTE*: Metallic trace elements

In this context, the present study examined the transfer of MTE at different interfaces of the Hafir forest to seepage water with quantification of MTE in vegetation (cork oak leaves and pinyon pine needles), surface litter, soils and infiltration water. The distribution and speciation of trace metal elements in the different solid phases of the environment were determined.

## Methods

**Figure 1:**
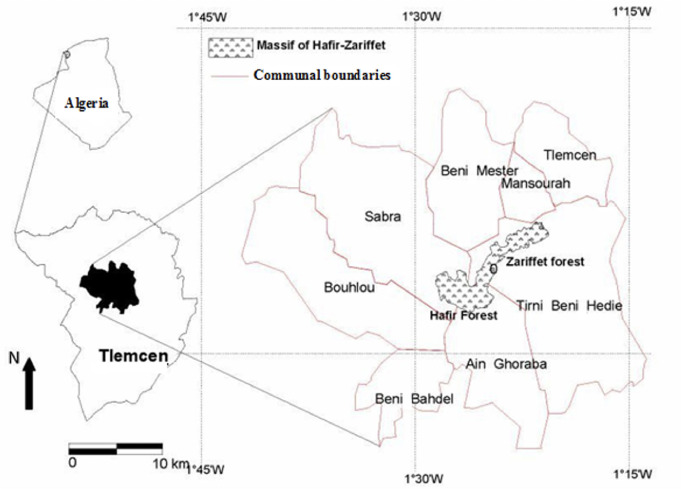
Location of Hafir forest^25^

The Hafir forest is located southwest of the city of Tlemcen. It contains the forests of Tlemcen, Maghnia and Tlemcen National Park *(Figure 1).* This forest extends over 1653 Ha and belongs to the communes of Ain Ghoraba (94 Ha) and Sabra (1559 Ha). The Hafir forest is located on a mountain massif, oriented from the east to the west at an altitude of 1000 to 1420 meters. The approximate distance from the nearest point of the forest to the main town of Tlemcen is 22 km.

The Hafir forest is characterized by a rugged relief. It has a high relative humidity and dense flora. The study station is located at an altitude of 1170 m and represents a plant recovery rate of 80%. The type of soil encountered in the forest territory is fersiallitic brown soil originating from limestone mother rock. It is a heavy soil poor in water reserves, but wet and porous and rich in bases, particularly Ca^+2^, Mg^+2^ and K^+^, existing under climatic vegetation (green oak and Aleppo pine) with a fairly dense undergrowth.^24^ It is a site which is representative of the context of contamination by atmospheric deposition. These soils are located along an axis which experiences high road traffic (500 vehicles/day).

### Sampling method

The analyses identified14 metals during two sampling periods in dry and wet weather, and the latter involved a rainy event characterized by runoff. Ten stations were distributed throughout the study area, following the distance from the road axis (0–1700 m). For each sampling point, an average sample was made from five elementary samples. At each station, zigzag sampling at 5 points, 0.5 m apart at a depth of 0–20 cm (Horizon A) was performed. At each point, a 500 g sample was withdrawn. Once collected, each sample was placed in freezer boxes with a polypropylene lid, labeled, stored at 4°C, and protected from light. The coordinates of the sampling points are shown in Table 1.

**Table 1:** Sampling Point Coordinates

**Distance from road**	**GPS coordinates**		

	Average altitude (m)	Latitude	Longitude
0 m	1254	34°46′35.94″N	1°25′58.37″W
2.5 m	1254	34°46′36.04″N	1°25′58.20″W
5 m	1254	34°46′36.15″N	1°25′58.30″W
10 m	1254	34°46′36.13″N	1°25′58.70″W
25 m	1254	34°46′36.59″N	1°25′59.08″W
45 m	1254	34°46′37.19″N	1°25′59.07″W
90 m	1257	34°46′38.39″N	1°25′59.97″W
260 m	1270	34°46′43.7″N	1°26′01.4″W
*600 m*	1227	34°47′08.1 ″N	1°25′52.6″W
*1700m*	1312	34°48′07.0″N	1°26′51.1″W

### Analysis protocol

Samples of 250 g of each specimen were dried in an oven at 40°C for 18 h, then sieved separately at 2 mm before being mixed and then ground in a ceramic mortar. The samples were then subjected to physico-chemical analysis: the measurement of granulometry (RO-TAP Steine Industrie), residual humidity, pH (HANNA, pH 211), electrical conductivity (WTW InoLab), resistivity, sulfur, sulphates, % C and organic matter content (% OM).

#### Determination of metals

For the metering of heavy metals, the samples were homogenized and then dried at 105°C +/− 5°C according to the AFNOR standard NF X31-102.^26^ The samples were then sieved to 2 mm (for elimination of plants, pebbles, etc.) according to the AFNOR standard NF X31-10, then surface sediments underwent two types of digestion: a total attack destroying the organic matter to determine the total concentrations of heavy metals, and a sequential extraction consisting of extraction of metals existing in different chemical forms in the sediments by successive washing with strong reagents.

#### Total contents of major elements and metallic trace elements

The AFNOR standard NF X31-147 was used to describe the dissolution of metallic trace elements by etching with hydrochloric (HCl) and nitric (HNO_3_) acids (commonly known as aqua regia) of the solid phases. This method obtained concentrations for analysis with atomic absorption spectrometry.

### Sequential extraction

In order to determine the speciation of the studied metals, the different phases of the sediment must be separated, and for the present study, the standard method of the Community Office of References was used.^27^ This technique is divided into three steps:

*Step 1*: Extraction of the water-soluble and acid-soluble fraction by acetic acid 0.11 M. This is the labile fraction which is easily exchangeable in an aqueous medium that is faintly acid and weakly complexing, it also includes metals bound to the carbonates.
*Step 2*: Extraction of the reducible fraction by hydroxylamine hydrochloride at 0.1 M. This is the less labile fraction; it corresponds to metals mainly bound to oxides, oxyhydroxides or hydroxides of iron or manganese.
*Step 3*: Double extraction of the oxidizable fraction with hydrogen peroxide at 8.8 M and the ammonium acetate at 1 M. It corresponds to metals bound mainly to organic matter and sulfides.

Table 2 shows the operating protocol used, to which we added a fourth extraction used in the majority of the sequential extraction schemes, the “residual phase”. Dosage of the metals in the extracted fractions was carried out by flame atomic absorption spectroscopy (FAAS). The device used was an atomic absorption spectrophotometer with an air/acetylene flame type (AURORA AI 1200). This is an elemental analysis method for the analysis of heavy metals in trace states. Elements absorb radiation where the wave length corresponds to those emitted during the return to the fundamental state of the atom.^28^

**Table 2:** Community Office of Reference Protocol for a Test Sample of 1 g of Sediment^29^

**Step**	**Reagents**	**Volume of solution (mL)**	**Temperature (°C)**	**Extraction time**
1	CH_3_COOH 0.11 M	40 mL	22 ± 5	16 h of agitation
2	NH_2_OH.HCl 0.1 M Acidified with HNO_3_ 2 M	40 mL	22 ± 5	16 h of agitation
3	H_2_O_2_ 8.8 M	10 mL	22 ± 5	1 h of agitation
			85 ± 5	1 h of agitation
		10 mL	85 ± 5	1 h of agitation
	NH_4_OAc 1M, (pH=2)	50 mL	22 ± 5	16 h of agitation

## Results

The present analyses estimated the total metal stock (mg/kg^−1^) at a given moment. This has the disadvantage of not providing any information about the mobility of metals in soil. Data on total content allows follow-up over time to detect diffuse and point-source contamination. The results of the analysis of the total concentration of metallic elements were compared with regulatory standards and are expressed in total contents.

In the present study, two sampling campaigns were carried out, the first at the beginning of the winter season (dry season) and the second in the rainy season after leaching by rainfall (wet season). Soil sampling was carried out at different distances from the road axis (0, 2.5, 5, 10, 25, 45, 90, 260, 600, and 1700 m). Two hundred and sixty meters (260 m) from the roads is an area of pinyon pine, a cork oak area at 600 m which has undergone several fires, and at 1700 m a sub-surface cavity which allows the infiltration of rainwater and is the highest point of this forest. Traffic in this area is light.

The total contents of metals and major elements in soil samples are presented in mg/kg^−1^ *(Tables 3 and 4),* by distance from the axis road and across the two sampling campaigns.

**Table 3:** Total Metal Contents of Soil Samples Across Seasons (mg.kg^−1^)

**Samples**		**Co**	**Ni**	**Mn**	**Cd**	**Cr**
0 m	DS	27*	18	172	5.2	73
	WS	105	150	120	3.5	28
2.5 m	DS	31.7	24.6	188	4.9	139
	WS	26	58	308	1.9	58.6
5 m	DS	45	28.2	107	4.4	49
	WS	58	14	440	39	49
10 m	DS	36	11	345	11	38
	WS	44	29	380	6	30
25 m	DS	70	18	129	5.9	54
	WS	260	195	260	3.9	38
45 m	DS	42	19	190	5.6	60
	WS	41	72	170	2.4	28
90 m	DS	49	9.8	202	3.7	21
	WS	51	76	230	1.5	42
260 m	DS	35	11	181	3.8	18.4
	WS	65	48	202	1.2	42
600 m	DS	22	23	376	4.4	23
	WS	43	30	523	2.5	46
1700 m	DS	17	139	31	61	342
	WS	52	160	41	26	566
***Threshold value^2^,^11^***		2	1–100	270	2	150
***Threshold value^32^,^33^***		23	50	300	2	150

^*^all units mg/kg

Abbreviations: DS, dry season; WS, wet season

**Table 4:** Total Metal Contents of Soil Samples Across Seasons (continued) (mg.kg^−1^)

**Samples**		**Pb**	**Cu**	**Zn**	**Fe**	**Ag**
0 m	DS	143	55	361	380	9
	WS	55	71	207	6880	10
2.5 m	DS	171	46	518	3412	11.9
	WS	62	23	364	4864	22
5 m	DS	96	63	367	3397	7
	WS	40	27	433	8300	4.6
10 m	DS	34	31	348	10323	32
	WS	102	15	342	8904	52
25 m	DS	113	38	387	8678	26
	WS	140	56	588	8456	14
45 m	DS	112	38	380	6922	39
	WS	140	15	275	6400	14
90 m	DS	56	35	414	8795	42
	WS	190	12.4	273	6547	2.4
260 m	DS	85	39	345	7563	29
	WS	106	15.4	254	7951	9.3
600 m	DS	126	38	368	8114	7.5
	WS	79.5	21	380	6822	5
1700 m	DS	451	4.3	14100	94	19
	WS	646	5.5	16682	64	14
***Threshold value^2^,^31^***		100	100	300	300	-
***Threshold value^32^,^33^***		100	100	1000	40000	-

Abbreviations: DS, dry season; WS, wet season

There are different references to estimate the level of contamination of soils with which to compare the present results. These references have been established according to particular soil uses, such as vegetable production and agricultural products linked to food.^30^

The present study compared the soil sample results with the AFNOR Standard NF U 44-041.^2^,^26^ The results of the metal assay, presented in Tables 3 and 4, indicate that the MTE contents can be highly variable within the same soil.

The results of the major element assay are illustrated in Table 5.

**Table 5:** Total Major Metal Contents of Soil Samples in mg.kg^−1^ Across Seasons

**Samples**		**Mg (mg.kg^−1^)**	**Ca (mg.kg^−1^)**	**Na (mg.kg^−1^)**	**K (mg.kg^−1^)**
0 m	DS	2560	798	578	116 000
	WS	9420	854	386	122 340
2.5 m	DS	3483	996	961	12 600
	WS	5639	1252	330	162 600
5 m	DS	2403	973	883	6000
	WS	7390	831	259	132 500
10 m	DS	1907	1094	1062	2734
	WS	4282	817	803	22 620
25 m	DS	2065	1350	1418	10 400
	WS	2097	831	635	3707
45 m	DS	1020	727	666	1841
	WS	1964	597	510	2560
90 m	DS	1536	1284	1060	2240
	WS	2117	784	666	3069
260 m	DS	1791	1206	963	6200
	WS	2797	690	666	2970
600 m	DS	2131	2280	1140	3524
	WS	2614	828	6240	3918
1700 m	DS	3712	1741	2996	9000
	WS	6860	1072	1624	18 971

Abbreviations: DS, dry season; WS, wet season

Table 6 reports the average results of the physicochemical characterization of soils in the Hafir study area during the two sampling campaigns (dry and wet seasons) and at different distances from the road axis. These soils are rich in organic matter and variation in organic matter over time was observed. Soil texture varied by distance from the road: sand (0–5 m), silt (5–10 m) and silt loam (25–45 m), silt (90–260 m) and sandy loam over 600 m.

**Table 6:** Physico-chemical Analysis of Soil Samples Across Seasons

**Sample**	**0 m**	**2.5 m**	**5 m**	**10 m**	**25 m**	**45 m**	**90 m**	**260 m**	**600 m**	**1700 m**
pH	8.50	8.43	8.37	7.88	7.68	7.20	7.32	7.32	7.195	8.30
χ (μS/Cm)	182.75	192.6	155.55	167.9	176.8	113.6	96.9	156.3	145.5	380.5
R (resistance) (Ω)	5500	5500	7000	6000	7000	9000	11000	6500	7000	5500
Sulfur (%)	1.88	1.74	2.10	1.98	2.38	1.44	2.22	1.84	2	1.255
Sulfate (%)	1.05	0.52	0.82	0.88	0.82	0.81	0.63	0.77	0.66	0.64
Sulfite (%)	0.17	0.77	0.52	0.4	0.76	0.23	0.32	0.39	0.58	0.52
C (carbon) (%)	0.92	0.46	1.53	2.84	2.09	1.03	1.1	2.09	2.82	2.83
OM (organicmatter) (%)	1.59	0.79	2.64	4.89	3.61	1.78	1.89	3.6	4.86	4.88
Moisture content (%)	13.64	15.56	19.91	21.6	24.20	19.22	16.80	25.42	24.11	15.36
Texture	Sandy loam	Sand loam	Silt	Silt	Silt loam	Silt loam	Silt	Silt	Sandy loam	Sandy loam

## Discussion

The results of the present study highlight the dispersion of metals, taking into account the major factors influencing the evolution of pollutant concentrations in the study area (road traffic, rainwater and runoff inputs, snow melt, dominant winds in the area and site morphology).The trace metal elements present in soil may originate from the pedo-geochemical background (natural phenomena)^30^ and can also result from direct influences by inheritance of the parent rock and bio-geochemical cycle or indirect effects of anthropogenic activities. The structure, texture and composition of soil confers special characteristics which influence the infiltration and retention of metallic trace elements.

### Cobalt

Total Co contents exceeded the AFNOR standard^26^,^32^,^33^ NF U 44-041 (23 mg.kg^−1^) in almost all samples taken during the two sampling seasons. Total cobalt contents fluctuated according to the distance to the road axis, particularly during the beginning of the wet season, where the contents are the highest. In the immediate vicinity of the road (0 m), the Co content was very high (105 mg.kg^−1^) and the maximum content was observed (260 mg.kg^−1^) at 25 m from the road. This content decreased rapidly moving away from the road for all the samples from the two sampling campaigns (dry and wet seasons).

### Chromium

All recorded values for Cr content were below the standard recommended by AFNOR *(Tables 3–4).*^26^,^32^,^33^ The highest concentrations were recorded in late winter (wet season) at distances close to the road. According to Juste *et al.*,^34^ Cr is considered to have very low mobility, and has both a natural and anthropogenic origin. Soils can also be enriched with Cr via the rejects of atmospheric depositions. Chromium may originate from dust emitted by catalytic converters in small amounts, as well as brake pads, clutch discs, and automatic transmissions.^35^

### Copper

The Cu content in Hafir forest soil was below the threshold recommended by the AFNOR standard (100 mg.kg^−1^).^2^,^26^,^31^ Overall, the Cu content in soil was higher in wet weather *(Table 4).* Soil leaching by rainwater transports pollutants to the soil, which may explain the increasing concentrations in the wet season.^36^ The highest concentrations of Cu 71 mg.kg^−1^ and 56 mg.kg^−1^ were detected directly beside the road and at 25 m in dry weather. Copper is widely distributed in nature and its average concentration in the Earth's crust is between 45 and 70 mg/kg according to Baize.^2^ Copper may originate from dust emitted from copper catalytic converters as well as brakes.^37^

### Iron

The Fe contents in Hafir forest soil were well below the threshold recommended by the AFNOR standard (40000 mg/kg^−1^).^26^,^32^,^33^ Iron is the most abundant metal in the sediments of Hafir and the distribution of this element was homogeneous across the study site. Iron enrichment is due to the geological context of the study area (fersiallitic soils). The richness of soil with iron originates in the nature of the substrate itself. Iron constitutes 5% of the earth's crust; it is released from rocks and is mainly soluble and easily mobilizable.^38^ Lubricants are a major source of iron.^39^ The highest concentrations of Fe were observed at 1700 m *(Tables 3–4).*

### Manganese

The total Mn contents fluctuated according to distance to the road axis. Manganese concentrations recorded in the dry season were higher than in the wet season, with the exception of two samples at 0 m and 45 m from the road. On the other hand, very high Mn values were recorded in the soil samples taken at 600 m and at the site located at 1700 m from the road *(Tables 3–4)* and far exceeded the standard recommended by AFNOR (300 mg/kg) for the two sampling campaigns.^26^,^32^,^33^

In addition, in dry weather at 2.5 m and 5 m, as well as at 10 m (for both samplings campaigns), the content of this metal at the edge of the road was 308 mg/kg and 440 mg/kg and 380 mg/kg and 345 mg/kg, respectively, which exceeded the AFNOR standard.

The temporal distribution of Mn concentrations showed a decrease with the infiltration of rainwater and the leaching of soil in wet weather. Manganese is the least abundant of the twelve major elements of the earth's crust (0.10). Manganese is derived from fuels containing MMT (methylcyclopentadienyl manganese tricarbonyl), an anti-knock substitute for lead.^40^

### Nickel

Very high levels of Ni were observed in the direct border at the road (0 m) and at 25 m from the road *(Table 3)* during the first sampling campaign. Antagonistic effects are observed between Ni on the one hand, and Cu, Zn, Fe on the other hand,^41^ as a high concentration of Ni in the medium decreases the absorption of nutritional elements.^42^ However, in the second season samples (wet season), the Ni contents were low and all were below the AFNOR standard (50 mg/kg). For Rousseau, the maximum ‘'normal” is 80 ppm, with an average of 40 ppm.^43^ Nickel originates from dust emitted by nickel catalytic converters.^37^

### Lead

Content fluctuations were observed for Pb during the two sampling campaigns. The Pb content exceeded the AFNOR standard at several points in the soil.^2^,^26^,^31^,^32^,^33^ This variability appears to be related to road traffic that contributes to the deposition of Pb in soils via dry and wet atmospheric depositions.^44^

The route of contamination is both direct through atmospheric deposition or indirect after the leaching of roads by rainwater.^45^ Lead generally binds to soil particles and other chelators; it is not carried to depth by leaching, resulting in a concentration gradient depending on depth.^46^ The highest concentrations of Pb are revealed at the beginning of the winter season (via dry fallout) at 90 m and at 1700 m from the road. It appears that the lead contents are not necessarily at the edge of the road. It also appears that, overall, Pb levels increased with distance from the road *(Table 3).* However, during the wet weather sampling season (wet weather deposition), and as a result of runoff and pavement leaching, the Pb concentration was higher at the roadside. The impact of road traffic is highlighted in the variability of Pb concentration in the forest soil.

### Zinc

Fluctuations in the total Zn content were observed during the two sampling campaigns. The values ranged from 207 mg/kg to 646 mg/kg *(Tables 3–4).* This latest content was measured, in dry weather, at a distance of 1700 m from the road. At 25 m from the road, a Zn content of 588 mg/kg was observed in the dry season. The soil can also be enriched by the anthropogenic contributions of urban activities and road traffic. It appears that brakes and tires are an important source of zinc, as well as lubricants.^47^

### Silver

Silver was observed in significant quantities at 10 m from the road and decreased gradually from 25 m up to 600 m. Its contents increased slightly at 1700 m from the road *(Tables 3–4).* Overall, the Ag content values were not significant, particularly in dry periods. In dry conditions, concentrations were high in the vicinity of road traffic. In wet weather, they were elevated moving away from the road (up to 10 m).

### Cadmium

The concentrations of Cd were higher in the soil in dry season as observed.^48^ This period allows for the accumulation of metals, and Cd contamination could be linked to the activity of plants. All Cd species of the soil solution are, a priori, absorbable by the plants.^49^ Absorption of Cd depends on the genus or variety considered also on the root absorption processes of passive absorption and jointly active absorption.^50^ The maximum Cd concentration (39 mg/kg) was observed at 5 m from the road.

Concentrations dropped starting from 10m to the minimum at 260 m (3.9 mg/kg and 1.2 mg/kg) respectively, in the wet and dry seasons *(Table 3).* We found that at the sampling point 25 m from the road, the Co, Ni, Pb, Cu, Zn content was high in the respective series. The sampling site was located on a slope of runoff *(Table 5),* which is marked by soil erosion. It has been determined that Cd is derived from lubricants.^51^

### Calcium and magnesium

The variability of Ca and Mg concentrations in forest soil was similar. On the one hand, Ca is necessary for crop development at the relief level and is present in significant quantities, particularly in wet weather *(Table 5)* and near the road, indicating that Ca can also arise from other major anthropogenic sources such as automobile traffic and long-range atmospheric transport of wind-induced dust.^52^

On the other hand, Mg was observed in higher quantities, especially in wet weather *(Table 5),* at distances close to the road.

For both sampling campaigns, Ca and Mg concentrations were notably lower moving away from the road axis. Moreover, the concentrations of Mg and Ca were higher in wet weather (after the rains). This can be explained by the fact that rainfall provides non-negligible amounts of elements, such as Ca and Mg. The origin of elements contributed by rains and enriching the soils has been the subject of several earlier studies.^53^,^54^

It was also observed that the content of Ca and Mg increased (slightly more in the case of Ca) in the soil located in the study station at 1700 m. Calcium was present at a high content (116000 mg/kg) along the roadside and in the dry season.

### Potassium and sodium

Potassium was found at all sampling points of the forest soil. The soil was richer in K at 600 m (at the site of the cork oak forest) and to a lower extent at 1700 m. Previous studies by Courtois and Masson^55^ have shown that the K content is an indicator of cork quality.

A maximum concentration (peak) of 6240 mg/kg in K was observed in the cork oak area during the sampling campaign after precipitation *(Table 5).* The high potassium content may be due to vegetation.^56^ For Na, a maximum concentration of 2280 mg/kg was observed in the cork oak plot in the dry season. It is possible that Na levels are derived from plant activity as well as the combustion of biomass and vegetation.^57^

### Physico-chemical characterization of forest soil

For pH, the results did not show significant changes during the two sampling campaigns. According to the pedological reference system (INRA 1995),^58^ the pH measured for soil at different distances from the road shows an alkaline character in the roadside (0–25 and 1700 m from the road) and neutral (pH between 6.5 and 7.5) at 45, 90 and 260 m from the road.

The elevations of pH at the roadside and at 1700 m can be attributed to the displacement of the carbonic equilibrium towards the formation of carbonates under the effect of photosynthesis, causing an increase in pH or a disturbance due to the emissions of pollutants.^59^ The low to moderately basic pH of the soil limits the mobility of metals and promotes their retention by soil particles.^60^ However, the large spatiotemporal variability of the total metal contents did not allow us to correlate the storage of the MTE with the soil pH. Inside the forest, the distribution of organic matter appears to be homogeneous and remains in the profile of forest soils. Organic matter content was particularly high (humiferous) in soils of the cork oak area at 600 m. Conductivity χ values fluctuated between 96.9 and 192.6 μS/Cm. However, at the station near the sub-surface cavity (1700 m from the road), the highest conductivity values were 380.5μS/Cm, as determined during the 1st and 2nd sampling campaigns, indicating high mineralization.

Metals are distributed in soils in various forms. They are found in exchangeable form in clays and organic matter which allows them to be absorbed by plants, as complexes or associated with organic molecules.^61^ They can be included in crystalline phases or directly adsorbed on particles of oxides or hydroxides of iron, aluminum and manganese.^62^

The form of metals in soils depends on their mineralogical composition, conditions of salinity, pH, redox, soil particle size, water content, the presence of ligands in solution or microorganisms. All these factors influence the solubilization of metals as well as their precipitation or adsorption.^63^,^64^ Interactions between the different soil compartments take place through the soil solution, which transports metals in all their forms, soluble or particulate. The form in which trace elements are present in the soil conditions their mobility and bioavailability.^65^

### Speciation of metallic trace elements and sequential extraction

The potential danger of the presence of MTE to the environment is directly related to the mobility of these elements, and therefore to the nature of the solid phases with which they are associated. Sequential extraction allows quantification of metallic elements in specific soil phases rather than total contents present in the samples. Elements can then be categorized as metastable or stable. Sequential extraction generally provides information on the distribution and fate of metals in the soil, and subsequently allows us to estimate their toxic potential. Analysis of the levels of Cd, Pb, Cu, Zn, Ni, cobalt (Co), iron (Fe), manganese (Mn), chromium (Cr), Ag and sodium (Na), calcium (Ca), magnesium (Mg), and potassium (K) in the Hafir forest area by the sequential extraction method was carried out on the samples of the two sampling campaigns (dry and wet seasons) at various distances from the road axis. The results of the sequential extraction analysis of the metal contents in the soil are presented for each metal (MTE or major element), in % in the form of histograms.

### Cobalt

Cobalt is mainly concentrated in the reducible phase for the two sampling campaigns *(Supplemental Material Figures 1–2),* while Co contents in the exchangeable phase are very low, particularly in dry weather. These results confirm the low mobility of Co. This observation has been noted by other authors.^10^ The affinity of Co to the different phases of soil is classified in the following order: dry and wet seasons: F2> F4> F3> F1.

### Chromium

By dry and wet deposition, Cr was mainly concentrated in the reducible phase *(Supplemental Material Figures 3–4)* and mainly on the roadside. This distribution is in agreement with previous studies that preferentially associate Cr to the reducible fraction,^66^ which means that Cr can be mobilized and become bioavailable and therefore directly influence the environment. In wet weather, it is present in the oxidizable phase at 25 m and 600 m from the road. Our observations are in agreement with Galan *et al.*, suggesting that the studied soils show contamination by Cr.^67^ This speciation of Cr (mainly associated with organic matter) is related to the richness of forest soil in organic matter at these points (3.61% and 4.86%), respectively. This observation has been previously reported.^68^ The classification in descending order of the phases is as follows: dry season: F2>F4>F1>F3 and wet season: F2>F4>F3>F1.

### Copper

At the roadside, most of the Cu occurred in the residual fraction (dry season) *(Supplemental Material Figures 5–6).* This proportion is consistent with that found in the literature,^69^ and therefore does not pose major environmental problems, except at 260 m where it is exchangeable (pinyon pine plot). In the second period, it was distributed among the 4 fractions mostly in the reducible fraction, thus at (25–90 m) in the residual fraction and in the exchangeable phase at 260 and 1700 m. The increase of Cu content in the organic phase has been previously observed,^70^ where the first two reducible and exchangeable fractions play an important role. Copper presents a potential risk to pinyon pine during both the dry and wet periods where it is concentrated in the exchangeable fraction and at the sub-surface cavity, but only in wet weather. Copper concentrations presented in the following order: dry season: F4> F2> F3> F1 and wet season: F2> F4> F1> F3.

### Iron

Iron was widely present in the residual phase *(Supplemental Material Figure 7–8)* and is characterized by low mobility. It is present in crystalline oxides and silicates.^70^ Unlike what one might expect a priori, the major fraction was the residual fraction and not the fraction representing the (reducible) elements of iron and manganese oxides with the exception at 2.5 m from the road after rainfall. This result indicates that a small percentage of Fe is in the form of an oxide or iron hydroxide such as goethite, ferrihydrite or hematite. The classification of the affinity of Fe to the constituents of the soil was as follows for the dry and wet seasons: F4> F2> F1> F3.

### Manganese

For both periods, the oxides of Fe or Mn represent the dominant phase of complexation *(Supplemental Material Figures 9–10).* It was exchangeable in the roadside (0–10 m) for the dry season and 90 m for the two seasons. In the light of this distribution of Mn, it appears that this metal exhibits average mobility. In this context, similar observations have been highlighted by several studies.^71^,^72^ The distribution of Mn is classified in the following sequence for the dry and wet seasons: F2>F1>F4>F3.

### Nickel

Nickel has a high affinity for the residual phase *(Supplemental Material Figure 11–12).* We also observed an average affinity for the reducible phase (in wet weather). Globally, it is weakly exchangeable, and therefore not very mobile. This observation is in agreement with that reported by a previous study.^73^ In contrast, the presence of Ni at 260 m from the road in the exchangeable phase and therefore mobile, may threaten the pine forest. But after runoff, it passes to the residual phase. The classification of the presence of Ni in the different fractions is presented in the following decreasing order for the dry and wet seasons: F4> F2> F1> F3.

### Lead

According to previous studies, naturally occurring Pb is mainly concentrated in the oxidizable and residual fractions,^74^–^77^ which is in agreement with the results of the present study *(Supplemental Material Figures 13–14),* since Pb is bound to the residual fraction, the oxidizable fraction, the exchangeable fraction and finally the organic fraction at the lowest level, reducing the possibility of the formation of very stable complexes in the presence of humic material. Indeed, Pb is known to be preferably associated with iron oxyhydroxides,^78^ which explains its surprising and significant proportion in the reducible fraction at the first point near the road (0 m). However, Pb was present in the exchangeable phase in the soil of the pinyon pine plot (260 m) during the dry and wet seasons. After the rains, cork oak soil (at 600 and 1700 m) had Pb contents in the exchangeable fraction. This cannot be without consequences for cork oak and infiltration water. Based on these results, Pb is potentially slightly mobile, with Pb soil pollution distributed as follows: dry season: F4> F2> F1> F3and wet season: F1> F2> F4> F3.

### Zinc

A previous study by Barona and Romero^76^ stated that the high content of Zn in sediments, and the fact that it is also present in residual form, implies that it does not pose a risk to the environment. Our results agree with these observations and those of Ma and Uren.^70^ Zinc was found to be primarily associated with the residual fraction and then distributed to different fractions through the different sampling points *(Supplemental Material Figures 15–16).*

A significant proportion of Zn was present in the exchangeable fraction at 5 m (in the dry season) and 10 m (in the wet season), in addition to the reducible fraction (at 45 m and 1700 m in dry weather). Zinc was distributed as follows: dry season: F4>F3>F2>F1 and wet season: F4>F3>F1>F2.

### Silver

Silver was present mostly in residual phases, possibly indicating a low level of risk *(Supplemental Material Figures 17–18),* while it was exchangeable at 10 m in dry weather. In wet depositions, Ag was present in the exchangeable phases along the roadside to 0 and 2.5 m, bound to the oxidizable fraction at 5 and 10 m, and bound to the reducible fraction at 260 m (pinyon pine site), and present in the residual phases in the cork oak plot and near the sub-surface cavity (1700 m). The distribution of Ag in the different phases occurred in the following order: dry season: F4>F3>F1>F2 and wet season: F4>F3>F2>F1.

### Cadmium

The results of the sequential extraction showed that Cd was present mainly in the residual phase *(Supplemental Material Figures 19–20)* and potentially mobilizable in wet weather and also observed in dry weather except for 0, 2, 5 and 1700 m from the road where the oxidizable fraction predominates, indicating that the majority of Cd is included in the alumina silicates, which has been reported by Andersen *et al.*^73^ On the other hand, Cd was present at 0 m, 2.5 m and 1700 m in the oxidizable phases, the Cd is linked to carbonates, from dry and wet deposition. Many studies indicate that Cd is preferentially associated with the carbonate fraction,^79^ and to the residual phase.^10^ By the dry deposits, Cd was exchangeable at 10 m and 25 m from the road, thus presenting a potential risk, but this effect disappeared after leaching of the soil at the end of the wet period, which can be explained by the runoff and the mobility of Cd to deeper horizons. Classification of the different fractions of Cd in order of predominance is as follows: dry period: F3> F4> F2> F1and wet period: F4> F1> F2> F3.

### Speciation of major elements

The results show that the Ca and Mg ions were relatively predominant in the carbonate phase and thus easily exchangeable *(Supplemental Material Figures 21–24).* The distribution was as follows: dry and wet seasons: F1> F2> F4> F3.

### Sodium and potassium

Sodium is exchangeable at 90 m for both periods, and in wet weather at 0, 10, 25, 260 m. The results showed that the Na is very present in the oxidizable phase respectively of the following order *(Supplemental Material Figures 25–26)*: dry season: F2>F3>F1>F4 and wet season: F3>F2>F1>F4.

For K, the results show that it was highly concentrated in the residual phase, consequently the potassium ion was almost immobile in the soil. It was concentrated in the exchangeable fraction at 2.5, 5, and 10 m of the road due to the atmospheric fallout linked to the road, and in wet weather it was exchangeable at 260 m (pinyon pine site). The distribution of K in the different phases occurred in the following order: F4> F1> F2> F3 *(Supplemental Material Figures 27–28).*

### Geochemical distribution of metallic trace elements and major metals in the studied soils

Knowledge of the total heavy metal content is necessary, but insufficient to evaluate their potential mobility and the resulting environmental risks. The prediction of these risks is closely linked to the physicochemical forms under which metals are present, namely their speciation. The study of the speciation of metallic pollutants provides information on their interactions with the solid phase, and on their binding forces with the latter, and therefore on their mobility. On the whole, the speciation study showed that the most important fraction is the residual fraction, which is not bioavailable for the medium.^80^ These results are consistent with the results of Tessier on the one hand,^20^ and the total contents according to AFNOR standards on the other hand, indicate that 70% to 80% of heavy metals are mainly bound to silicates and in the residual fraction. In addition, 12–29% are present in stable forms, and less than 2% of the metals could be bioavailable. Thus, the ten heavy metals studied have almost identical distributions in each of the four sedimentary phases.

## Conclusions

Natural and anthropogenic disturbances in the studied forest ecosystem can lead to daily intakes of metallic trace elements in the surrounding soil. These inorganic micropollutants are fluently encountered on sites polluted by polycyclic aromatic hydrocarbons whose speciation is influenced by the organic matter dissolved in soil. The MTE assay results indicate the spatial and temporal variability of total MTE contents in forest soil in the present study.

The impact of road traffic is reflected in the variability of the concentration of several trace elements in forest soil, such as Co, Mn, Ni, Zn, Pb, Ag, and Cd. They were primarily observed at very high levels on the roadside and by dry atmospheric depositions. However, the geological context is also highlighted by the presence of Fe and Cr. Soil erosion seems to be the origin of the soil enrichment, with Co, Ni, Pb, Cu, and Zn observed primarily 25 m from the road. The contribution of rainfall to the enrichment of the soil with major elements such as Ca and Mg was also indicated. Conversely, the deep roots of forest vegetation play a role in the depletion of soils from major elements such as calcium.

The speciation of each metallic trace element in the soil of the surrounding environment is influenced by certain factors such as road traffic, dry atmospheric deposition and wet fallout, and intakes of rainwater and runoff which influence the evolution of the speciation of these pollutants and consequently their mobility and associated potential risk.
