# Supplementary material for: Chemical Speciation and Potential Mobility of Heavy Metals in Forest Soil Near Road Traffic in Hafir, Algeria
Source: J Health Pollut. 2021 May 28;11(30):210614. doi: 10.5696/2156-9614-11.30.210614 (PMC8276720; doi:10.5696/2156-9614-11.30.210614)
Supplement: Supplementary file 1 [file Benhachem_Supplemental_Material.docx]

**Supplemental Material**

**Figure 2: Speciation of Co (%)**

**(wet season)**
